# Multiple pulmonary sclerosing pneumocytoma, based on a study of 36 cases worldwide

**DOI:** 10.1038/s41598-024-63185-7

**Published:** 2024-05-28

**Authors:** Pan He, Jianwei Wang, Jiong Guo, Shunqi Li, Weidong Zhang

**Affiliations:** 1 Department of Cardiothoracic Surgery, Henan Provincial Chest Hospital (Chest Hospital of Zhengzhou University), Zhengzhou, Henan China; 2 Henan Provincial Chest Hospital, Room 1, Weiwu Road, Zhengzhou, 450003 Henan China

**Keywords:** Pulmonary sclerosing pneumocytoma, Multiple tumor, Treatment and diagnosis, Respiratory tract diseases, Surgical oncology, Risk factors, Oncogenesis, Pathology

## Abstract

To analyze the clinical characteristics and to improve clinicians’ understanding of multiple pulmonary sclerosing pneumocytoma (PSP) patients. A total of 36 PSP patients with multiple tumor characteristics were identified from the literature search. They were compared with 43 solitary PSP patients diagnosed and treated in our hospital in the past 5 years. Thus, the pathogenesis, clinical symptoms, diagnosis methods, treatment strategies, and prognosis of pulmonary sclerosing pneumocytoma (PSP) patients with multiple tumors were explored. Patients with multiple PSP are mostly distributed in Asia (88.89%) and are females (83.33%). PSP can be located in any one lobe (19.44%), or grow across ipsilateral lobes (44.44%), or even, bilateral lobes (36.11%). It can be accompanied by metastasis (9.09%) and is prone to misdiagnosis (27.78%). Compared with solitary PSP, the occurrence age of multiple PSP was younger (mean ± standard deviation [SD]: 40.36 ± 18.12: 51.28 ± 12.74 years), but there was no significant difference in sex, tumor size (mean ± SD: 43.54 ± 46.18: 30.56 ± 17.62 mm), or symptoms. Individualized surgical resection is required for treatment, including pneumonectomy (17.65%), lobectomy (23.53%), subpulmonary lobectomy (38.24%), or combined lobectomy (5.88%). Multiple PSP is relatively rare. Surgical resection within a limited time should be the main treatment for such patients. The prognosis of patients with multiple PSP is generally good, but inappropriate diagnosis and treatment plans may lead to poor prognosis.

## Introduction

In 2015, the World Health Organization officially named primary pulmonary sclerosing hemangioma as pulmonary sclerosing pneumocytoma (PSP), and classified it into pulmonary adenoma^1^. Most scholars believe^2,3^ PSP to mostly occur in Asian women. Its overall incidence is low and shows solitary nodules with single, quasi-round, and occasional calcification on imaging. Few cases of multiple and metastatic PSP have been reported. However, in recent years, several literature works have reported PSP with multiple characteristics. In 1980, Indian scholar Joshi^4^ first reported a 40-year-old female patient with multiple pulmonary nodules in both lungs, and later, confirmed it as multiple PSP. Since then, at least 35 patients with multiple PSP have been reported by scholars worldwide^5–31^.

The location of multiple PSP in these patients differs: some are located in one lung lobe, some in multiple lobes of the unilateral chest, and some grow across the chest in bilateral multiple lobes. Compared with general PSP, multiple growth is a special manifestation of PSP, and their etiology, diagnosis, and treatment have certain specificities. A total of 36 PSP patients with multiple growth were searched through the network, and the medical records of 43 solitary PSP patients who underwent surgery in our hospital in the past five years were collected. In this study, the clinical characteristics of these two types of patients are compared and analyzed. In addition, the clinical characteristics of multiple PSP patients and the appropriate diagnosis and treatment methods are explored to provide clinical diagnosis and treatment references for more medical workers.

## Materials and methods

“Pulmonary sclerosing hemangioma, multiple” and “pulmonary sclerosing pneumocytoma, multiple” are considered as the keywords, respectively, through the China National Knowledge Infrastructure, Wanfang, Google Scholar, and PubMed databases. A total of 36 cases of PSP patients with multiple characteristics are retrieved and these characteristics are summarized in Table 1^4–31^.

**Table 1:** Comparison of patients’ characteristics with multiple and solitary PSP.

	Multiple PSP		Single PSP		P value
	n	%	n	%	
Published time (n)	36		43		
Before 2000	3	8.33			
2000–2005	5	13.89			
2006–2010	3	8.33			
2011–2015	6	16.67			
2016–2020	19	52.78			
Distribution area (n)	36		43		
Asia	32	88.89	38	100.00	
Other	4	11.11			
Gender (n)	36		43		0.1974
Male	6	16.67	12	27.91	
Female	30	83.33	31	72.09	
Age (year) (n)	36		43		0.0035
Mean ± SD	40.36 ± 18.12		51.28 ± 12.74		
Quartile	37 (25, 54.5)		52(43, 60)		
Range	16–73		24–74		
Diagnosis (n)	34		43		
Percutaneous lung biopsy	6	17.65			
Intraoperative pathology	4	11.76			
Postoperative pathology	24	70.59	43	100.00	
Tumour size (mm) (n)	28		43		0.1649
Mean ± SD	43.54 ± 46.18		30.56 ± 17.62		
Quartile	27.5 (20, 50)		27(20, 36)		
Extrapulmonary metastasis (n)					
Yes/no	3/33		0/43		
Misdiagnosis (n)	10	27.78	0		
Pulmonary tuberculosis	4	11.11			
Lung metastases	4	11.11			
Others	2	5.56			
Therapy (n)	34		43		
Sublobar resection	13	38.24	16	37.21	
Sublobar resection + lobectomy	2	5.88			
Lobectomy	8	23.53	27	62.79	
Pneumonectomy	6	17.65			
Observation	5	14.71			

*PSP* pulmonary sclerosing pneumocytoma.

Simultaneously, 43 cases of solitary PSP in patients from our hospital were used as the control group. The differences between solitary and multiple PSP were compared by analyzing their general characteristics, symptoms, treatment methods, tumor characteristics, and other information.

### Data presentation

Numerical variables of normal distribution were displayed as mean ± standard deviation, and those of nonnormal distribution were displayed as median (interquartile range). Categorical variables were displayed as frequency (percentage).

### Ethics approval and informed consent

This study was conducted in accordance with the principles of the Declaration of Helsinki, and the study protocol was approved by the Ethics Committee of the Henan Provincial Chest Hospital. Written informed consent has been provided by our hospital patients to have the case details. And another part of patients’ information in this article comes from the references.

## Results and discussion

In 1956, Liebow and Hubbell^5^ first described the predecessor of PSP: pulmonary sclerosing hemangioma. In 2015, the World Health Organization formally renamed it as PSP^1^. It is composed of “two cell types, four patterns”, that is, two cell kinds of cuboidal surface cells and rounded cells, and four histology areas of papillary, solid, sclerosing, and hemorrhagic areas. PSP is a rare disease; especially, in those patients with multiple lesions, it is easy to be misdiagnosed as metastatic tumor, pulmonary tuberculosis, and other diseases^5–10^.

**Figure 1:**
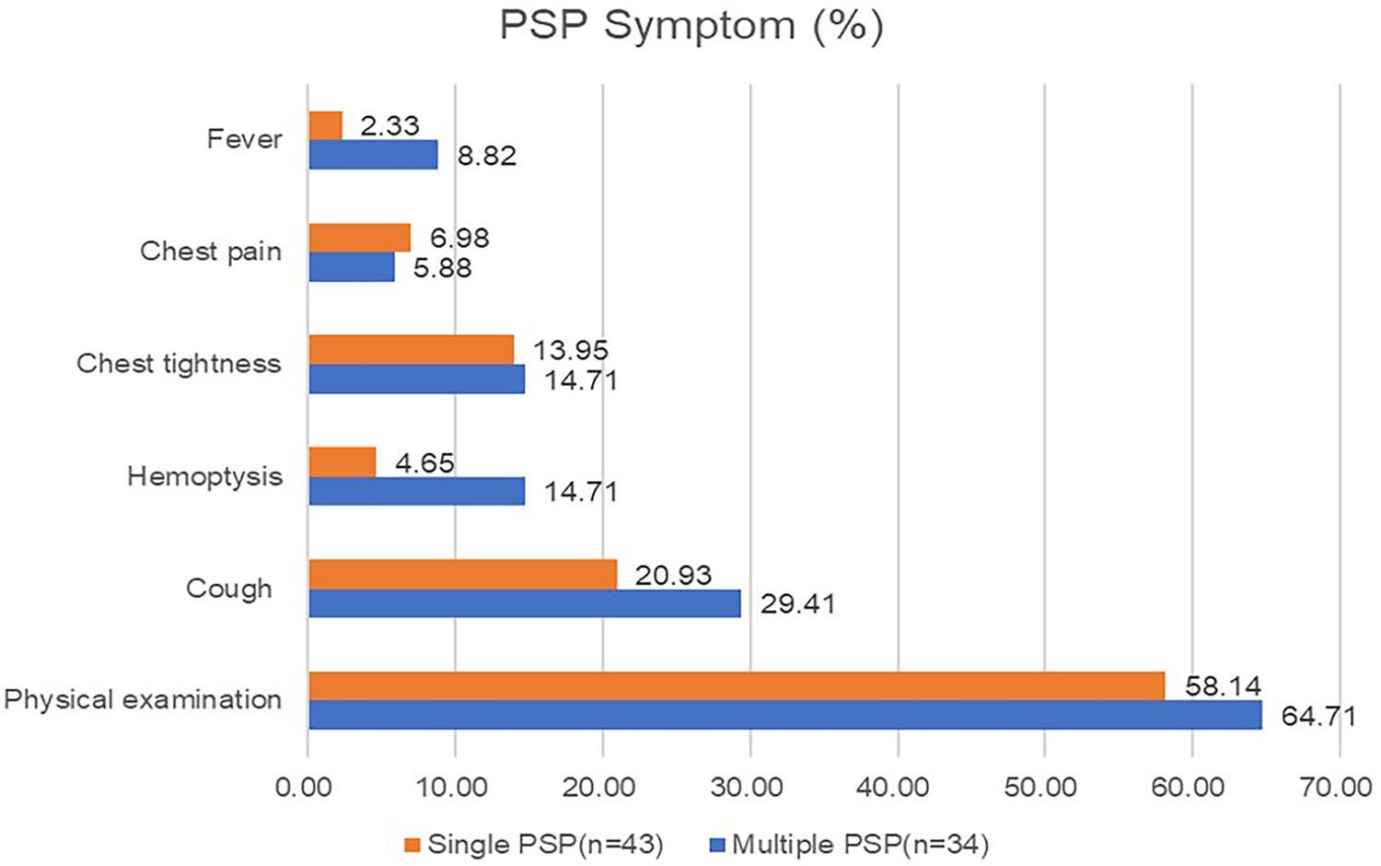
The symptom of multiple PSP and single PSP.

Table 1 indicates that since the first detection of multiple PSP patients in 1980, only a small number of subsequent cases have been reported. Among them, the number of reported cases in the past five years has been higher, accounting for more than half (52.78%). Most of them are distributed in Asian countries (88.89%), such as China (47.22%), Japan (25.00%), South Korea (8.33%), India (8.33%), and also, in Europe and America (11.11%), which is similar to the solitary PSP patients. There is no significant difference between the two groups^11–13^. Multiple PSP mostly occurs in women (83.33%) within the age range of 16 to 73 years (median [interquartile range]: 37 [25, 54.5]; mean: 40.36 years), of which, a relatively large proportion is of the age group 21–30 years (27.78%). Compared with the solitary PSP group, those patients in the multiple PSP group were younger (mean: 40.36:51.28 years). Most of the patients (64.71%) were accidentally found by physical examination, while a small number sought medical attention because of symptoms such as “cough (29.41%)”, “hemoptysis (14.71%)”, chest tightness (14.71%)”, “chest pain (5.88%)”, and “fever (8.82%)”. During the hospital visit, a computed tomography (CT) examination of the chest revealed multiple pulmonary nodules that were diagnosed as multiple PSP. There was no significant difference in the occurrence probability of various symptoms between the multiple PSP group and the solitary PSP group. This means multiple PSP did not have specific symptoms (Fig. 1).

**Figure 2:**
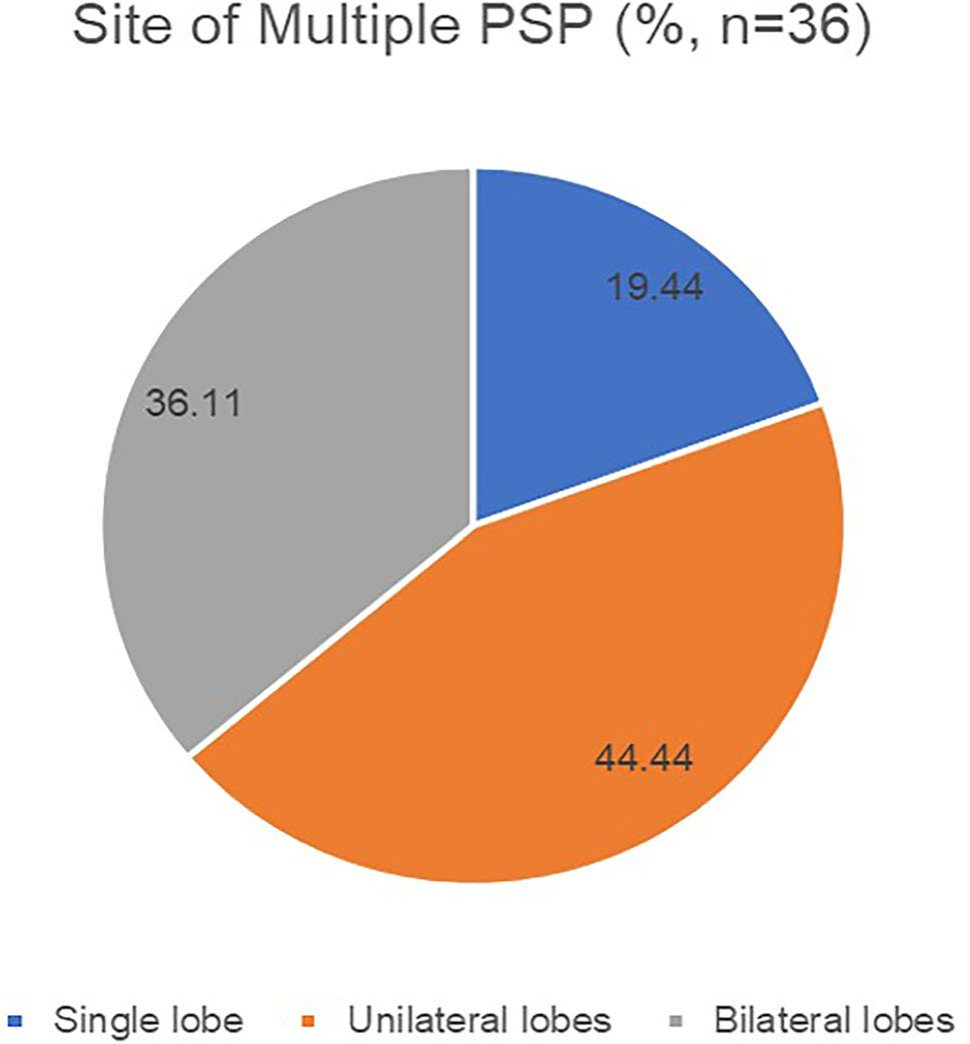
The site of multiple PSP.

**Figure 3:**
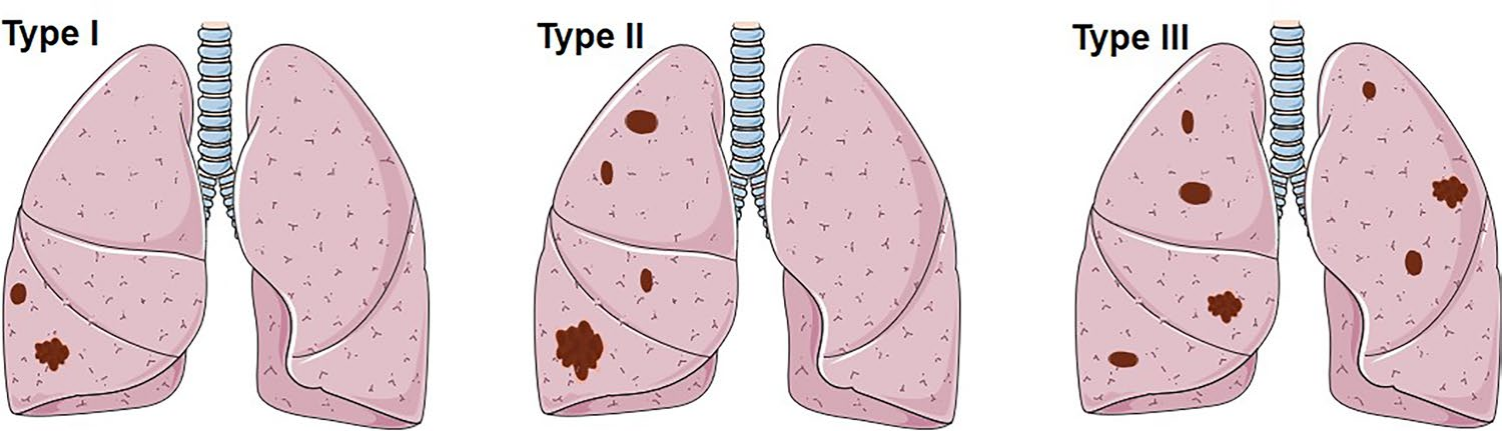
The subtypes of multiple PSP. Type I: multiple PSP located in a single lung lobe; Type II: multiple PSP located in multiple lung lobes of the unilateral chest; Type III: multiple PSP located in multiple lung lobes of the bilateral chest.

Patients with multiple PSP generally require a CT examination of the chest for its detection. Imaging analysis of 36 patients with multiple PSP revealed the maximum lesion diameter to range from 6 to 226 mm (median [interquartile range]: 27.5 [20, 50] mm; mean ± SD: 43.54 ± 46.18 mm). This is larger than the average diameter of solitary PSP patients (mean: 43.54:30.56 mm), but there is no statistical difference between the two groups. With regard to the distribution of tumor locations, some scholars^14^ classified them into five different subtypes, such as “Type I: a dominant SP (Sclerosing pneumocytoma) with satellite nodules that occur in one lobe (multi local); Type II: SPs that are distinct, separate fractions in the same love; Type III: SP occurring in different ipsilateral lobes; Type IV: SP occurring independently in central lobes and Type V: SP occurring bilaterally in all lobes”. After analysis, this classification method is considered to be relatively cumbersome and to have no clinical guiding significance in diagnosis and treatment. Therefore, the distribution of tumor locations is simplified into three types: I, multiple PSP located in a single lung lobe; II, multiple PSP located in multiple lung lobes of the unilateral chest; and III, multiple PSP located in multiple lung lobes of the bilateral chest (Figs. 2, 3). An analysis revealed that type II multiple PSP was more, approximately 44.44%, followed by type III multiple PSP (36.11%), whereas type I multiple PSP was relatively less (19.44%). This classification method can better guide the clinical treatment strategy of multiple PSP. An analysis of the imaging findings of multiple PSP revealed 8.33% of patients to have lymph node metastasis or even extrapulmonary metastasis^15^, which was significantly higher than the lymph node metastasis rate of solitary PSP^17–20^.

**Figure 4:**
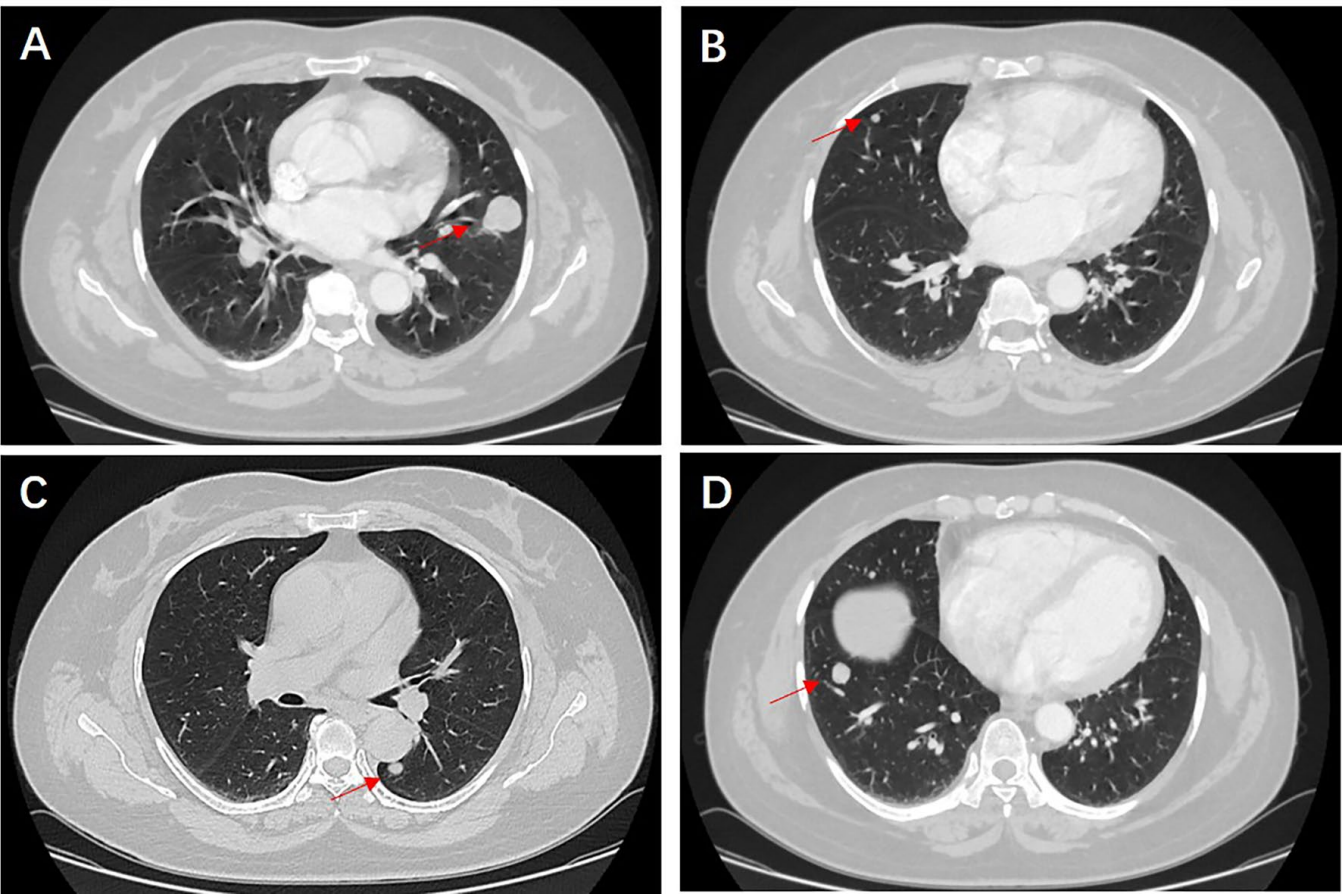
Chest CT of multiple PSP. This is a rare case of multiple PSP. The patient’s physical examination revealed the presence of multiple nodules in both lungs. A chest CT scan confirmed the existence of a total of 4 circular pulmonary nodules, distributed as follows: 1 in the upper lobe of the right lung, 1 in the lower lobe of the right lung, 1 in the upper lobe of the left lung, and 1 in the lower lobe of the left lung. The largest nodule, measuring 26 mm in diameter, was surgically excised from the left upper lobe and subjected to pathological analysis, which confirmed the diagnosis of PSP. Consequently, the final diagnosis was determined to be multiple PSP, and the patient is currently undergoing follow-up care.

Figure 4 demonstrates that multiple PSP is not image specific; hence, multiple PSP diagnosis by a CT examination of the chest alone is difficult. Some patients with a previous history of malignant tumors might first consider the diagnosis of pulmonary metastases when they find multiple round nodules in the lungs. They may give up treatment at this time, but some reports^6–8^ suggest that multiple PSP can be diagnosed by puncture or surgical biopsy. Patients with multiple pulmonary nodules, who seek medical attention due to fever, can be misdiagnosed to have pulmonary tuberculosis by inexperienced doctors. Even if their tuberculosis-related tests are negative, they might still receive unnecessary anti-tuberculosis treatment^5,9^. However, some reports^21^ imply that patients with multiple PSP complicated with pulmonary tuberculosis may also exist. For patients who have multiple round pulmonary nodules with a previous history of malignant tumors, the time of discovery of pulmonary nodules is important. Similar to most scholars’ reports^14–16^, the diagnosis of multiple PSP is mainly based on the pathological diagnosis of postoperative resected specimens. The accuracy rate of percutaneous lung biopsy pathology and intraoperative rapid pathology diagnosis is low. Therefore, for multiple round pulmonary nodules which are difficult to be diagnosed, the authors propose to promptly perform a minimally invasive surgery for nodule resection and pathological diagnosis.

It is still controversial whether multiple nodules of multiple PSP are multiple primary lesions or metastatic lesions. Many scholars^23–26^ have conducted relevant research in the past, but so far there is no meaningful analysis result. And Jung^28^ discovered all germline variations in the two PSP nodules to be the same. Whereas somatic cell variations were mutually exclusive when he conducted somatic mutation sequencing and pathway analysis of nodule lesions in a patient with multiple PSP. Therefore, Jung perceived that they had different origins and belonged to multicentric lesions. The authors discovered only 8.33% of patients with multiple PSP to have lymph node metastasis (refer Table 1). The death of a patient with multiple PSP was reported^15^. Multiple lesions gradually enlarged and fused into a huge mass during the 10-year follow-up. In this study, multiple PSP is considered as multiple primary lesions, but these are only conjectures at present. Sufficient pathological and genetic analysis data to confirm this is still scarce.

In accordance with the current literature data^28,29^, multiple PSP is rare and grows slowly; but, it is not a malignant tumor, and the prognosis is mostly good. Although the prognosis of most multiple PSP is good, there are still some reports^6,30^ that suggest the growth of multiple PSP over one lung lobe or one side of the chest, which even compresses the surrounding blood vessels, trachea, and heart, leading to death^15,29^. There are also some reports^8,20^ that imply the relief of symptoms (fever, hemoptysis) in patients only after the complete removal of multiple nodules. An analysis of the existing literature data suggests lobectomy for multiple PSP located in a single lung lobe (type I); For patients with multiple PSP located in multiple lung lobes of the unilateral chest (type II), the largest tumor should be removed, or if the tumor is large and close to fusion, total pneumonectomy should be considered; For patients with multiple PSP located in multiple lung lobes of the bilateral chest (type III), if the tumors are small, they can be observed. However, for larger tumors, individualized treatment options such as lobectomy, multi-site wedge resection, stereotactic radiotherapy, and radiofrequency ablation can be chosen. For patients undergoing surgical treatment, lymph node dissection can also be considered. The evaluation of lymph node metastasis has a certain guiding significance for prognosis. The aforementioned treatments still lack medical evidence because of the less number of patients with multiple PSP and the availability of long-term follow-up clinical data. Hence, continuous study on this matter is required to confirm them.

This study was subject to certain limitations. Notably, the sample size of patients with multiple PSP was relatively small, and their distribution was widely dispersed. Consequently, the authors were only able to analyze the characteristics of PSP patients with malignancy based on globally reported data. While some conclusions were drawn, the absence of multicenter clinical research verification remains a notable deficiency. Nevertheless, the authors maintain that our research findings can still serve as a valuable reference for clinicians and offer potential avenues for future investigation.

## Conclusion

Multiple PSP is a rare type of PSP that occurs mostly in middle-aged women in Asia. PSP can be located in any lobe of the lung, and can grow across the lobe of the lung, or even across the chest. Most scholars consider multiple PSP to be multiple primary lesions, but their pathogenesis is still unknown. Its treatment should be individualized according to the location of multiple nodules, but larger lesions should be promptly removed. The prognosis of patients with multiple PSP is generally good, but if not treated in time, especially for patients with large tumors or multiple PSP with metastasis, it can lead to serious consequences and even death.

## Data Availability

The datasets used and analysed during the current study available from the corresponding author on reasonable request.

## Abbreviations

CT: Computed tomography
PSH: Pulmonary sclerosing hemangioma
PSP: Pulmonary sclerosing pneumocytoma
SP: Sclerosing pneumocytoma
